# Decoy bypass for appetite suppression in obese adults: role of synergistic nutrient sensing receptors GPR84 and FFAR4 on colonic endocrine cells

**DOI:** 10.1136/gutjnl-2020-323219

**Published:** 2021-06-03

**Authors:** Madusha Peiris, Rubina Aktar, David Reed, Vincent Cibert-Goton, Ausra Zdanaviciene, Writaja Halder, Adam Robinow, Simon Corke, Harween Dogra, Charles H Knowles, Ashley Blackshaw

**Affiliations:** 1 Centre for Neuroscience, Surgery and Trauma, Blizard Institute, Barts and The London School of Medicine and Dentistry, Queen Mary University of London, London, UK; 2 Gastrointestinal Diseases Research, Queen's University, Kingston, Queensland, Canada

**Keywords:** obesity, appetite, gut hormones, neuroendocrine cells

## Abstract

**Objective:**

Colonic enteroendocrine cells (EECs) store and release potent anorectic hormones that are key regulators of satiety. EECs express multiple nutrient sensing receptors, particularly for medium-chain fatty acids (MCFAs): GPR84 and FFAR4. Here we show a non-surgical approach with targeted colonic delivery of MCFA, which induces EEC and neuronal activation leading to anorectic effects.

**Design:**

A randomised, double-blind, placebo-controlled, cross-over study was performed in obese adults given combined GPR84 and FFAR4 agonists in colonic release capsules before meals. We measured serum hormones, energy intake and appetite perception. Cell type, activation by agonists and hormone/serotonin release were determined in human colonic explants. Mouse colonic afferent nerve responses to nutrients/mediators were recorded electrophysiologically.

**Results:**

Subjects receiving GPR84 and FFAR4 agonists had reduced overall calorific intake and increased postprandial levels of PYY versus placebo. Receptors including GPR84 and FFAR4 were coexpressed on human colonic EEC. Activation of GPR84 exclusively induced intracellular pERK, whereas FFAR4 selectively activated pCaMKII. Coactivation of GPR84 and FFAR4 induced both phosphoproteins, and superadditive release of GLP-1 and PYY. Nutrients and hormones convergently activated murine colonic afterent nerves via GLP-1, Y2 and 5-HT3 receptors.

**Conclusions:**

Colonic GPR84 and FFAR4 agonists reduce energy intake and increase postprandial PYY in obese adults. Human colonic EECs coexpress these receptors, which activate cells via parallel intracellular pathways and synergistically evoke hormone release. Further synergism occurs in sensory nerve responses to MCFA and EEC mediators. Thus, synergistic activation of colonic endocrine cells via nutrient receptors is an important target for metabolic regulation.

**Trail registration number:**

NCT04292236.

**Significance of this study**

**What is already known on this subject?**

- The most effective current treatment for obesity and type 2 diabetes is gastric bypass surgery, but it is restricted in availability, irreversible, costly and may cause harm. Shunting of undigested food (including medium chain fatty acid (MCFA)) to the distal gut triggers hormone release by activating enteroendocrine cell (EEC) via specific nutrient sensing receptors.
- Boosting colonic levels of the bacterial product propionate increases hormone release and reduces energy intake in humans, further implicating the colon as a site for metabolic regulation.

**What are the new findings?**

- Administering a colonic release formulation of MCFA (found normally only in the upper gut) before meals reduced food intake and boosted plasma PYY in obese adults without adverse effects.
- MCFA cooperatively stimulate nutrient receptors, which synergise through different intracellular pathways in EEC to maximise hormone release. This action on colonic EEC leads in turn to synergistic activation of afferent nerves projecting to the central nervous system (CNS).

**How might it impact on clinical practice in the foreseeable future?**

- The stimuli used in this study were safe and unrestricted for human use; furthermore, side effects were absent.
- Later phase clinical trials in obesity could begin soon after refinement of capsules. We also now understand the biology of an important pathway for regulation of energy intake.

## Introduction

Gastric bypass surgery, Roux-en-Y gastric bypass (RYGB) in particular, is currently the treatment of choice for obesity and associated type 2 diabetes.^1^ It achieves weight loss and glucose tolerance via several mechanisms including delivery of food to the terminal ileum and colon, where previously naïve nutrient receptors on enteroendocrine cells (EECs) are activated by a wide range of undigested nutrients, ultimately resulting in increased circulating levels of the potent anorectic hormone PYY^2^ and the insulinotropic hormone GLP-1. In addition, RYGB alters microbial populations leading to changes in luminal short-chain fatty acid and bile acid concentrations, which also bind to nutrient or bile acid receptors expressed on colonic EECs, collectively contributing to reduced food intake.^3 4^ Physiologically activated colonic EECs that are capable of responding to a multitude of nutrient stimuli may therefore represent an important target for decreasing food intake via peripherally regulated mechanisms.

Energy intake, appetite and satiety are controlled by several sensory, mechanical and psychological factors with both short-term and long-term effects. EECs are one important molecular regulator of appetite as they store and release orexigenic (eg, ghrelin) and anorexigenic hormones (eg, GLP-1, PYY and CCK)/transmitters (5-HT) that balance feelings of hunger and satiety.^5–10^ These hormones exert their effects on central pathways such as the appetite centre in the arcuate nucleus that contain AgRP/POMC neurones that respond to neuroendocrine signals leading to reciprocal inhibition of appetite increasing/decreasing pathways.^11 12^ The close apposition of EECs to nerve endings in the gut mucosa also permits local effects of hormones on afferent neurons before these rapidly degrade after release due to effects of enzymes such as dipeptidyl peptidase-IV (DPPIV).^13^

EECs, expressed from stomach to rectum, are traditionally classified according to hormone/peptide content, although emerging evidence suggests hormonal expression shifts during stages of cell maturation.^14–16^ Interestingly, the distal region of the gut has a high density of EECs including GLP-1 and PYY containing L cells and 5-HT containing enterochromaffin (EC) cells.^17^ We have previously shown that nutrient-sensing G-protein coupled receptors (GPCRs) binding to specific amino acids and short/medium/long-chain fatty acids, including GPR84 and FFAR4, are expressed at high levels in the colon.^18^ Due to the variety of nutrient receptors and paucity of EECs relative to colonocytes, it could be reasoned that EECs must respond to multiple stimuli in a concurrent manner to reflect the multinutrient content of human diets. Indeed, costimulation of GPR119, FFAR4 and GPR40 induces synergistic GLP-1 release from mouse colonic crypt cultures^19^ suggesting synergy of nutrient sensing GPCRs may exist in human EECs. We focused on FFAR4 and GPR84 as we and others have shown they: (A) are highly expressed in the colonic region and (B) respond to agonist stimulation by releasing anorectic hormones.^18 20^

The development of new effective medical treatments for obesity will require delineation of molecular mechanisms that underpin the normal physiological processes of nutrient-mediated appetite regulation. Sumithran *et al* 21 elegantly demonstrated that even following successful weight loss within an 8-week timeframe using calorie restriction diets, the anorectic hormone profile of these subjects does not change, even after 52 weeks. This suggests that long-term maintenance of weight loss requires targeting of EEC physiology to boost anorectic hormone release.

The aim of this study was to determine how dual nutrient stimulation may impact EEC activity using in vivo, ex vivo and clinical trial methodologies. The current study, a proof-of-concept trial in obese volunteers, demonstrates that coadministration of colonic GPR84 and FFAR4 agonists decreases food intake and increases PYY release in active versus placebo. We show that the underlying cellular mechanism is the expression of multiple nutrient sensing receptors on EECs that when stimulated concurrently, synergistically boosts cell activation and anorectic hormone release. Using mouse neuronal recordings, we demonstrate that peripheral afferent neuronal activity is subsequently increased in response to nutrient content and EEC activity.

## Methods

### Ethical approval

All subjects provided informed, written consent to participate. It was also registered with www.clinicaltrials.org.

### Patient and public involvement

Volunteers for the clinical trial were recruited from the general public after newspaper coverage of the study. Patients undergoing resection were asked to participate in the study on the day of surgery, while biopsies were collected from patients visiting the Endoscopy Unit, both at the Royal London Hosptial, Whitechapel. All patients were told of the types of studies that were being carried out and how their contribution would benefit understanding of essential gut processes. Patients were not involved in the study design but were informed that results would be available by contacting reserachers directly (as per guidance on patient information sheet given to all participants) as well as in open access peer review publications.

### Proof of concept obese volunteer study

We hypothesised that colonic delivery of endogenous GPR184 and GPR124 agonists would reduce food intake and increase circulating levels of PYY and GLP-1. The predefined primary outcome was change in calorific intake, and secondary outcomes included changes in levels of circulating hormones, PYY, GLP-1 and ghrelin. Men and women aged 18–60 years with a body mass index (BMI) of 30–40 kg/m^2^ were invited to participate. Potential subjects were excluded if they had previous gastrointestinal surgery, major health problems and/or were taking medication for type 2 diabetes.

#### Study design

The study used a double-blind, randomised, placebo-controlled, cross-over design. Twenty subjects were randomised to receive either active treatment or placebo with a 4-week washout period to account for menstrual fluctuation that can influence appetite with sample size calculated based on previous studies examining similar outcomes to colonic nutrient treatment.^22 23^ Prior to each study visit, subjects were asked to fast overnight from 20:00. At visit 1, weight and height were recorded, and BMI was calculated. At the beginning of each visit, subjects were cannulated via the antecubital fossa to collect blood samples (3 mL) at baseline (t=0) and 30 min intervals until t=480 min (8 hours). Prior to each blood sample being taken, subjects were asked to record their levels of hunger, satiety and fullness using 100 mm visual analogue scales (VAS).^24^

#### Active and placebo

Active capsules contained a total of 500 mg 3′3 diindolylmethane (DIM) (250 mg, Olympian Labs), 2100 mg alpha linolenic acid (ALA) contained within perilla oil (500 mg, 90 LiCaps, Fairvital) and 2400 mg lauric acid (LA) (Sigma Aldrich). Note: DIM (10 mM) and ALA (100 mM) both stimulated human ex vivo colonic tissue in Ussing chambers leading to activation of pERK and pCaMKII pathways, respectively (data not shown). Visually identical placebo capsules were filled with methylcellulose (Sigma). All capsules were coated with Phloral (Intract Pharma Ltd, UK) to ensure capsule delivery and disintegration in the colon. Colonic arrival and release of contents coated with Phloral has been demonstrated in volunteers as described by Varum *et al*.^25^

The first set of capsules was given 60 min prior to breakfast (time-point 0 hours) and the second 60 min before lunch (time-point 5 hours). The maximum calorific intake for breakfast was 903 kcal and lunch was 1340 kcal, and subjects were asked to eat as much as they desired up to this maximum. Following completion of the meal, the amount of food consumed was quantified and calorific intake calculated. All study visits commenced between 08:00 and 09:00 and were conducted at the Wingate Institute of Neurogastroenterology. Throughout the visit, the nurse/investigator maintained regular communication with the subject to encourage good compliance.

#### Circulating hormone assay

Prior to blood collection, EDTA Vacutainer tubes were cooled on ice. Immediately after blood collection, 10 µL of DPPIV inhibitor (50 Nm), fluoxetine (100 Mm) and moclobemide (100 Mm) were added to prevent hormone/5-HT degradation. Plasma was collected by centrifugation (10 min at 1000×g), and 1 mL aliquots were stored at −20°C. Total GLP-1, total PYY, active ghrelin and gastric inhibitory polypeptide (GIP) were quantified using a human multiplex kit according to manufacturer’s instructions (Milliplex MAP Multiplex assay, Merck Millipore). 5-HT was measured using an ELISA (BA E-5900, Labor Diagnostika Nord) as previously described.^18^

### Human colonic tissue collection

Full thickness samples of human ascending colon were obtained from 4 to 6 patients (five male, median age 53 year, range 31–64 years) undergoing surgery for colon cancer at The Royal London Hospital (Barts Health NHS Trust). All specimens were obtained from patients with non-obstructive tumours that were not occuring in the context of inflammatory bowel disease. Specimens were taken with the permission of the histopathologist following macroscopic examination and were a minimum of 10 cm away from tumour, resection margins or lymphatic drainage field.

### Immunohistochemistry

Following surgical resection, tissues were fixed in 4% paraformaldehyde (4°C overnight). Tissues were cryoprotected in 30% sucrose/PBS then mounted in optimum cutting temperature medium. Tissue sections of 10 µm were cut on a Cryostat (Leica CM1860), incubated with blocking buffer (Dako, UK) for 1 hour, before primary antibodies were applied overnight (4°C). Antibody details are provided in online supplemental methods. Tissues were washed (PBS; 3×5 min), and species-specific secondary antibodies were conjugated to Alexa Fluor fluorescent dyes (1:400, Thermo Fisher Scientific, UK) applied for 1 hour, before washing (PBS; 3×5 min), mounting (Vectashield hard set mounting media, Vector Laboratories, USA) and cover-slipping. Controls with no primary antibody were used in all experiments to check for non-specific secondary antibody binding. Leica DM4000 epi-fluorescence microscope was used to visualise immunoreactivity of sections. To ensure uniformity of acquired images, all sections were orientated and cut in the same manner. Images were captured using MetaMorph software (Molecular Devices, UK) and prepared for figures using Photoshop (Adobe) and PowerPoint (Microsoft).

10.1136/gutjnl-2020-323219.supp1 Supplementary data

### Ussing chamber experiments

Human colonic mucosal sheets (from cancer resections) were divided into 1×1 cm segments, and each segment was mounted in an Ussing flux chamber. We used a method developed by our group for optimised nutrient stimulation of human colonic mucosal sheets.^18^ Briefly, the luminal surface was exposed to 10 mL nutrient solution, while the basolateral surface was exposed to 10 mL Krebs solution, for 20 min. All solutions were carbogenated and maintained at 35°C–37°C. Colonic mucosa was then fixed in 4% paraformaldehyde overnight.

### Ex vivo human tissue hormone release assays

As previously described,^18^ human colonic mucosa was dissected into small, approximately 0.5 mm blocks, and each specimen was weighed before incubation with 250 µL of carbogenated solutions of buffer (control) or nutrients in a 96-well plate for 15 min (5-HT assay) or 20 min (hormone assay) at 37°C. A customised tissue culture medium with 4.4 mM L-glutamine and 6 mM glucose was used in order to ensure cells were healthy but not stimulated with nutrients. Inhibitors were used and hormone/transmitter levels were evaluated as described above.

### Extracellular electrophysiological recordings of mouse proximal colon

Nerve bundles running alongside mesenteric blood vessels that innervate the proximal region of the mouse colon were identified under a dissecting microscope and drawn into a borosilicate glass suction electrode (Harvard Apparatus, UK) prefilled with Krebs buffer. These comprise both vagal and splanchnic afferent nerves, which were both therefore recorded. However, activation by nutrients and gut hormones is lacking in most studies of spinal afferents,^26 27^ suggesting that the afferent fibres we studied are predominantly vagal. Nerve activity was recorded on a Neurolog headstage (Digitimer Ltd, UK), amplified at a gain of 5000×, band pass filtered (100–2000 Hz) and data acquired (20 kHz sampling rate; micro1401, Cambridge Electronic Design, UK) to a desktop computer running Spike2 (Cambridge Electronic Design, UK) software. Action potentials were counted using an online spike processor (Digitimer Ltd, UK), and the threshold level for spike processing was set to the smallest identifiable spike (typically ~100 µV).

### Data analysis

For cell counting following immunohistochemistry experiments, five fields of view at 40× objective, covering the entire section so as to remove image acquisition bias, were analysed from 4 to 6 individual tissue samples. Cells clearly immunoreactive positive, found within colonic crypts and captured completely in field of view, were counted as previously described.^18^ The number of full, unbroken crypts in each field of view was counted to normalise cells/crypt in nutrient stimulation experiments assessing pCaMKII and pERK. Differences between counted cells per sample were assessed for statistical significance using Prism software and p<0.05 was considered significant.

In electrophysiology experiments, analysis was performed offline using the spike sorting function of Spike2 to discriminate afferent nerve activity of individual units.^28^ Units were considered as responders if the increase in action potential discharge during the 5 min luminal nutrient perfusion was greater than 20% the baseline firing (measured during the 5 min preceding nutrient perfusion).^29^ Data were displayed as mean±SEM.

A sample size of 20 subjects was estimated based on calorific intake data from a previous study.^23^ To analyse VAS questionnaires, recorded answers for each time point in active versus placebo was averaged and compared using a two-way analysis of variance and Sidak’s multiple comparison test. Changes in total calorific intake for active and placebo conditions (relative to time 0) were analysed using paired Student’s t-test, and a p value of<0.05 was deemed significant. Area under the curve (AUC) for GLP-1, PYY, 5-HT and ghrelin was calculated and compared between active and placebo and analysed using paired Student’s t-test. Data were deemed normal following D’Agostino-Pearson omnibus normality test and are presented as mean±SEM. A p value of <0.05 was considered significant. All data were analysed using GraphPad Prism V.8 software.

## Results

### Effect of combined GPR84 and FFAR4 agonist supplementation in proof-of-concept study

**Figure 1:**
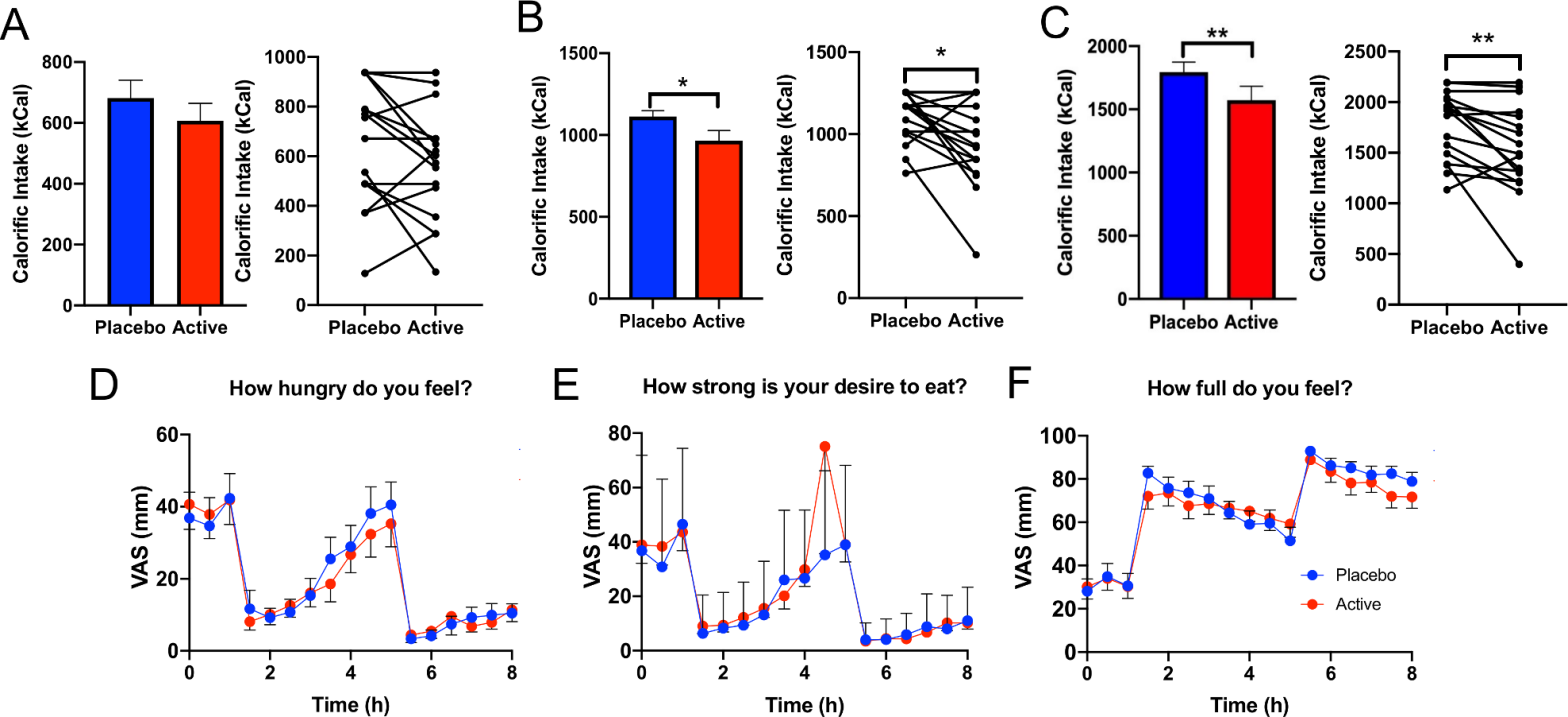
Colonic delivery of nutrient combination to obese subjects significantly reduces calorific intake. (A) Calorific intake at breakfast was unchanged between active versus placebo treatment. (B) Calorific intake at lunch was significantly lower in active versus placebo treatment (p=0.019). (C) Total calorific intake (including calorific intake from breakfast and lunch) was significantly lower in active versus placebo treatment arms (p=0.008). (D) VAS scores assessing feeling of hunger at each time-point in active versus placebo were unaltered. (E) VAS scores assessing desire to eat was not different between active versus placebo. (F) VAS scores assessing fullness showed no difference between active versus placebo. VAS, visual analogue scale.

Twenty obese human volunteers who met inclusion criteria (BMI: 30–40, no major gut surgery, not a type 1 or type 2 diabetic) were recruited to the study (mean BMI: 34.2±0.46 cm/kg^2^ and mean age: 48.9±2.0 years). To assess the effect of combined GPR84 and FFAR4 agonist stimulation, we used 3′3 diindolylmethane and LA as natural GPR84 agonists and perilla oil (containing 60% alpha-linolenic acid) as a natural FFAR4 agonist. No adverse effects, including nausea or vomiting, were reported by the 20 volunteers when given the active or placebo capsules. Targeted colonic delivery of these compounds significantly reduced calorific intake of the lunch meal (placebo: 1113 kCal, active: 965.7 kCal; p=0.0193) and overall calorific intake (breakfast and lunch meals combined (placebo: 1794.5 kCal, active: 1572.7 kCal, p=0.008), a 13.3% and 12.4% mean reduction (compared with placebo), respectively (figure 1B, C). No change in calorific intake was observed of the breakfast meal (figure 1A). Interestingly, there was no difference in subjective ratings of appetite such as feelings of hunger, desire to eat and fullness between the active and placebo treatments (figure 1D–F).

**Figure 2:**
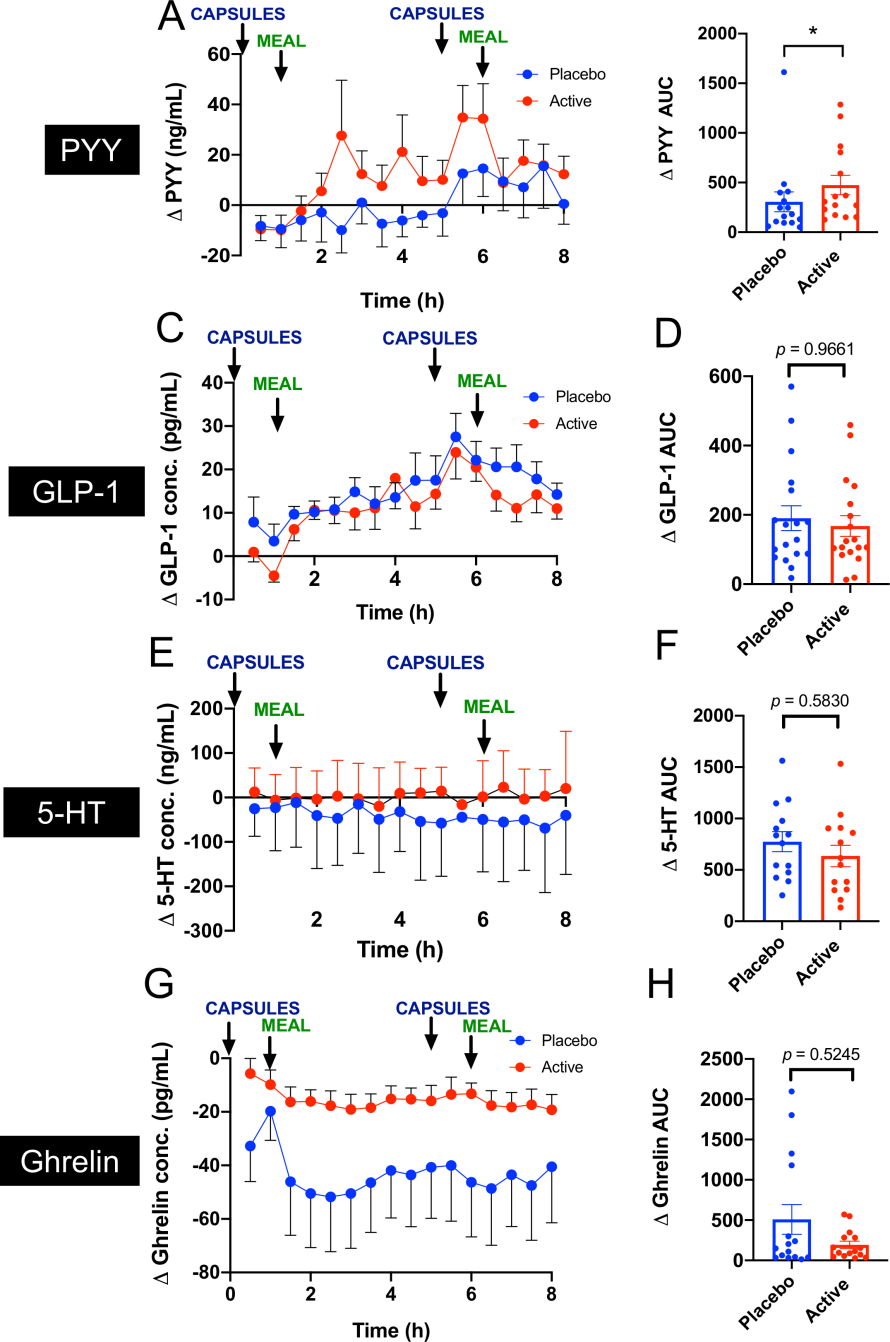
Effect of acute coadministration of 3′3 diindolylmethane (DIM), alpha linolenic acid and lauric acid (LA) on gut hormone levels in obese subjects. (A) Circulating levels of PYY were significantly increased in the active treatment compared with placebo. (B) GLP-1 levels did not significantly differ between active and placebo. (C) 5-HT levels did not significantly differ between active and placebo. (D) Ghrelin levels did not significantly differ between active and placebo. Capsules were given at time 0 and 5 hours, and breakfast was given at 1 hour and lunch given at 6 hours as indicated by arrows.

Circulating levels of appetite regulation hormones were assessed as a physiological end-point throughout the study day during both active and placebo stimulations. Coadministration of GPR84 and FFAR4 agonists significantly increased circulating levels of total PYY compared with placebo (active: AUC_∆_ 474.9 min × pg/mL vs placebo: 305.7 min × pg/mL, p=0.018; figure 2A). Increased PYY was sustained from 3 hours, when capsules arrive and begin to disintegrate in the colon (figure 2A).^25^ Active GLP-1 levels were not significantly changed following nutrient treatment (active: AUC_∆_ 190.5 min × pg/mL vs placebo: 167.9 min × pg/mL, p=0.9661; figure 2B), nor were circulating levels of 5-HT (active: AUC_∆_ 635.7 min × ng/mL vs placebo: 774.4 min × pg/mL, p=0.5830; figure 2C). We also assessed ghrelin levels that did not significantly change in active treatment versus placebo (active: AUC_∆_ 191.9 min × pg/mL vs placebo: 508.4 min × pg/mL, p=0.5245; figure 2D). The basal levels of the hormones PYY (placebo: 89.4±27.7 pg/mL, active: 94.8±36.4 pg/mL), GLP-1 (placebo: 12.9±2.9 pg/mL, active: 14.7±3.3 pg/mL), 5-HT (placebo: 107.8±10.2 pg/mL, active 101.3±26.4) and ghrelin (placebo: 57.1±21.1 pg/mL, active: 22.9±5.4 pg/mL) were not different between visits.

### Expression of nutrient sensing receptors on human EECs

**Figure 3:**
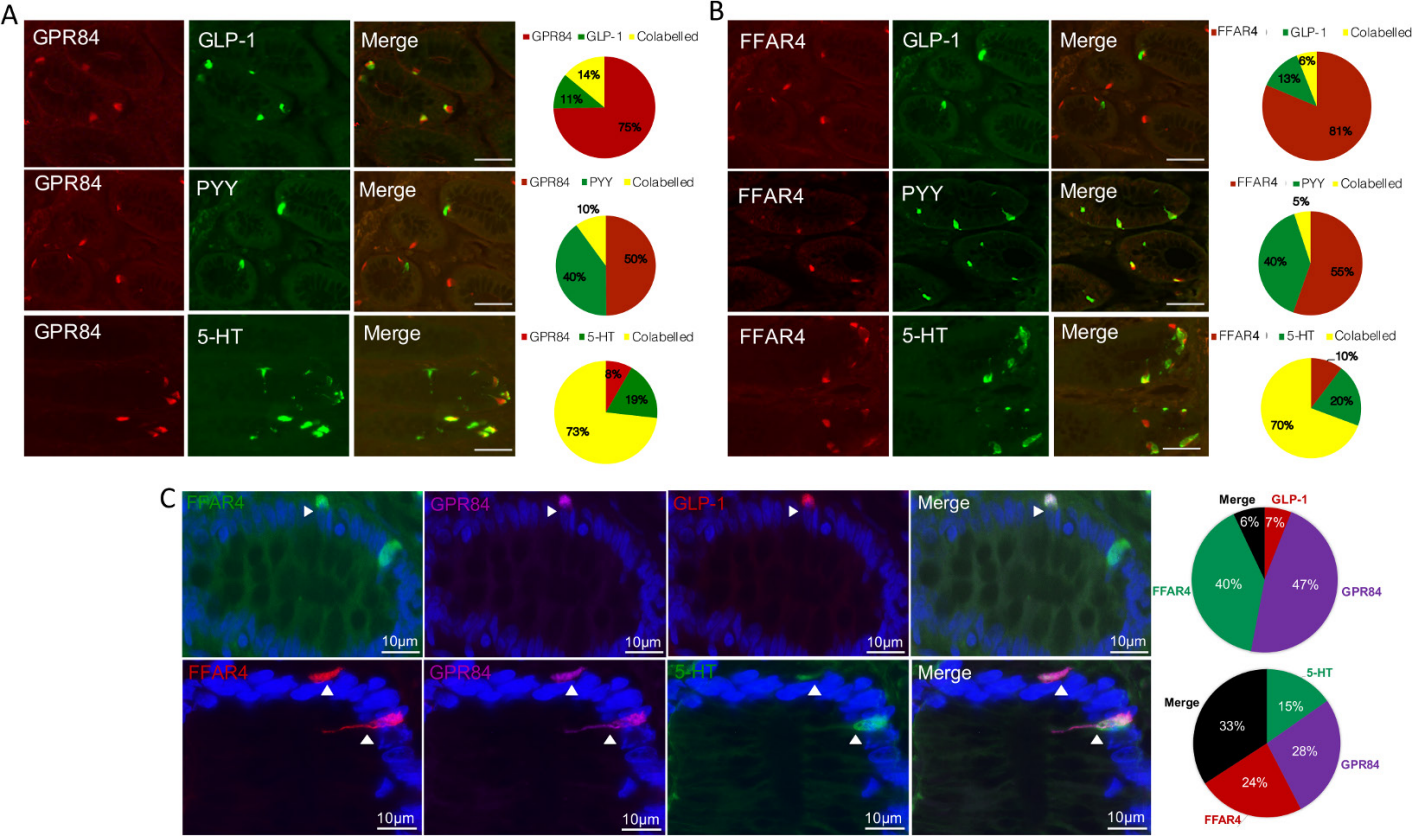
L cells and enterochromaffin (EC) cells coexpress GPR84 and FFAR4 in human proximal colon. (A) GPR84 is expressed on GLP-1/PYY containing L cells and 5-HT containing EC cells in human proximal colon mucosa. Expression of GPR84 was highest in EC cells, with a smaller population of L cells expressing this GPCR. n=5. (B) FFAR4 expression was observed on both GLP-1 and PYY containing L cells, although in a small subpopulation. EC cells, however, expressed FFAR4 in majority of 5-HT containing cells. n=5. (C) Top panel: FFAR4 and GPR84 are coexpressed on GLP-1 containing L cells, as shown in the merged image. Analysis of coexpression is shown in the graphs where cells were analysed for expressing GLP-1 alone, FFAR4 alone, GPR84 alone and when all three were found expressed on the same cell as shown by ‘merge’. n=5. Bottom panel: FFAR4 and GPR84 are coexpressed on 5-HT containing EC cells. Analysis of coexpression is shown in the graphs where cells were analysed for expressing 5-HT alone, FFAR4 alone, GPR84 alone and when all three were found expressed on the same cell as shown by ‘merge’. n=5.

To investigate nutrient sensing GPCRs on native human EEC, we obtained fresh, macroscopically normal proximal colonic epithelium from surgical resections. Immunohistochemistry revealed that fatty acid receptors GPR84 and FFAR4 were located on EEC; L cells and EC cells (figure 3A, B), as was the short chain fatty acid (C2-C7) receptor GPR43 and peptone/amino acid receptor GPR92 (online supplemental figure 1A, B), confirming our previous mRNA expression data along the human gut.^18^ The great majority of EEC on which GPCRs were found were EC, since they were 5-HT immunoreactive, whereas the minority were L cells, which coexpressed GLP-1 and PYY (figure 3A, B, online supplemental figure 1A, B). GPCR colocalisation studies showed that the long-chain fatty acid receptor FFAR4 was coexpressed with the medium-chain fatty acid receptor GPR84 on both L cells and EC cells (figure 3C). The colonic population of EC cells outnumbered L cells 5-to-1.^17^ The degree of GPR120/GPR84 coexpression observed in EC cells was greater, 33% vs 6%, respectively (figure 3C). FFAR4 also colocalised with the peptone receptor GPR93 on both EEC cell types (online supplemental figure 1D).

10.1136/gutjnl-2020-323219.supp2 Supplementary data

### Activation of nutrient receptors on human EECs

**Figure 4:**
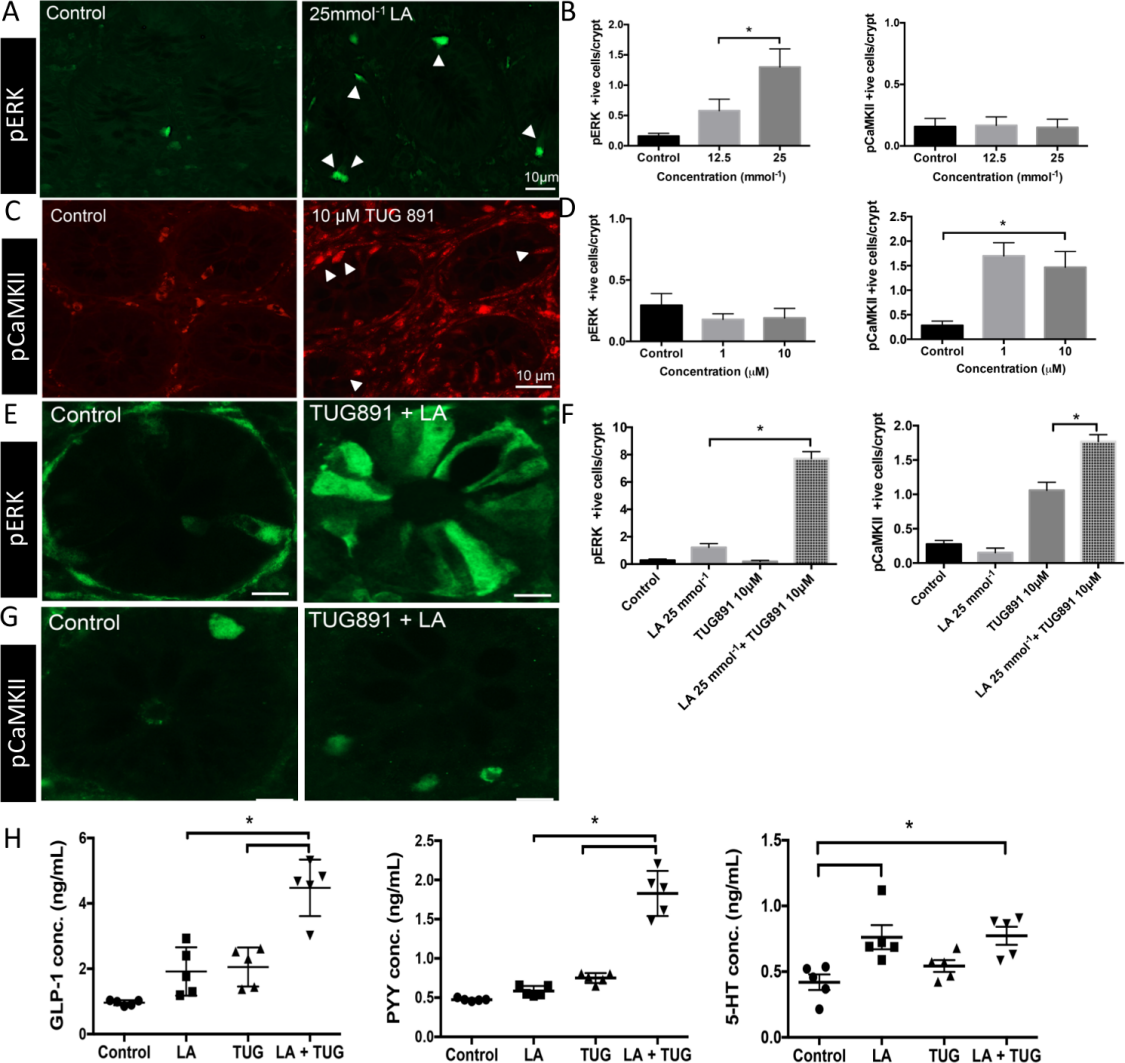
GPR84 and FFAR4 agonists activate separate intracellular pathways that converge to boost release of GLP-1 and PYY. (A) Stimulation of human colonic mucosa with GPR84 agonist, lauric acid (25 mmol^−1^) increases PERK expression in human colonic epithelial cells (green) compared with control buffer. n=4. (B) Lauric acid stimulation specifically induces expression of PERK (in a concentration dependent manner) and not pCaMKII, in human colonic mucosa. n=4. (C) FFAR4 agonist TUG891 (10 µM) increases expression of pCaMKII in human colonic epithelial cells (red) compared with control buffer. n=4. (D) TUG891 stimulation specifically induces expression of pCaMKII (in a concentration dependent manner) and not PERK, in human colonic mucosa. n=4. (E) Coapplication of lauric acid (25 mmol^−1^) and TUG891 (10 µM) enhances cell activation as measured by PERK immunoreactive cells. n=5. (F) quantification of cells positive for PERK showed co-application of Lauric acid (25 mmol^−1^) and TUG891 (10 µM) results in fourfold increase of positive cells compared with control and single nutrient application. co-stimulation doubled the number of pCaMKII cells compared with single nutrient application. n=5. (G) Immunoreactivity for pCaMKII increased in colonic crypts compared with buffer stimulation in human colonic mucosa. n=4. (H) Coapplication of lauric acid (25 mmol^−1^) and TUG891 (10 µM) to human mucosal tissue doubles the release of anorectic hormones GLP-1 and PYY compared with stimulation with single nutrient solution. Release of 5-HT is not enhanced by coapplication of GPR84 and FFAR4 agonists. n=5.

We determined if activation of nutrient receptors led to activation of phosphoprotein pathways in human EEC by exposing the luminal surface of colonic mucosal sheets to selected nutrients in an Ussing chamber. This revealed induction of pERK by the selective GPR84 ligand LA (C10, 25 mM) (figure 4A, B)^30^ but not by the potent and selective FFAR4 agonist TUG-891 (10 µM)^31^ (figure 4F). Conversely, pCaMKII was induced by TUG-891 but not by LA, indicating a high degree of selectivity of phosphoprotein pathways for each GPCR (figure 4C, D‒F). We have previously demonstrated that GPR84 stimulation by LA induces pERK expression in cells including L and EC cells.^18^ Here we show that TUG891 acting on FFAR4 induces pCaMKII expression in L and EC cells in addition to colonocytes (online supplemental figure 2A, B). When colonic mucosal sheets were simultaneously exposed to ligands of both receptors, expression of pERK and pCaMKII was seen in the same specimen (figure 4E‒G).

10.1136/gutjnl-2020-323219.supp3 Supplementary data

This raised the question of whether different intracellular pathways may contribute synergistically to boost EEC responses to nutrient receptor activation. Incubation with different nutrient stimuli led to different patterns of EE mediator release from colonic biopsies. LA activated release of total PYY, active GLP-1 and 5-HT (figure 4H) but not release of ghrelin and GIP (data not shown). TUG-891 alone released only PYY and GLP-1 (figure 4H). Strikingly, in the in vitro study, the combination of TUG891 and LA specifically boosted release PYY and GLP-1 to between two and four times the value for each individual nutrient but did not improve 5-HT release (figure 4H).

### Downstream effects on mouse colonic afferent nerves

**Figure 5:**
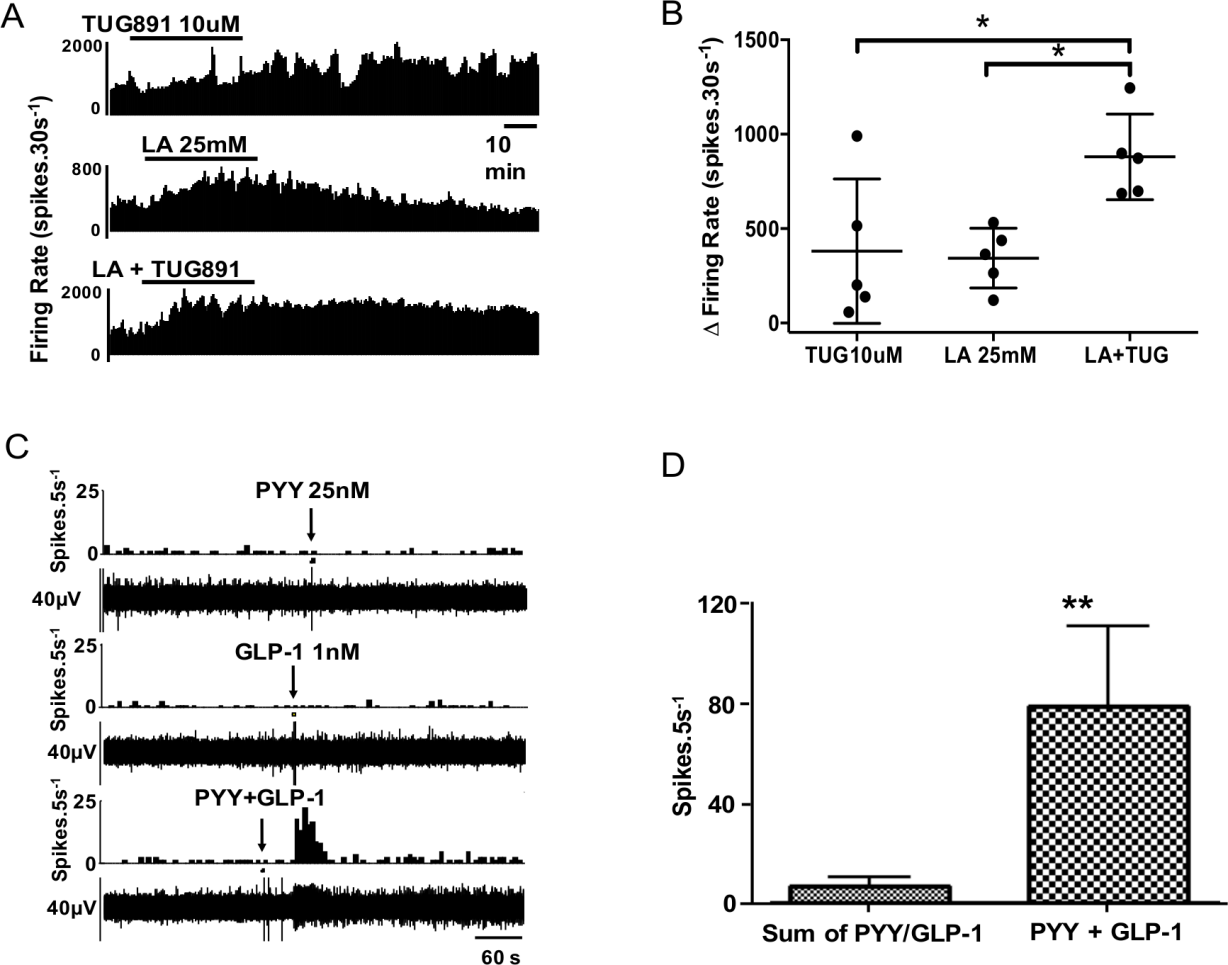
Synergistic effects are exerted on proximal colonic afferents by nutrients and appetite regulating hormones. (A) TUG891 evoked a slow-onset response in neuronal firing, whereas lauric acid (LA) had a faster onset effect with greater potency. (B) Combination of TUG891 and LA evoked a synergistic effect on afferent nerve firing rate. n=5/treatment. (C) Example rate histogram and RAW neurogram records of an afferent unit response to PYY (1 µM) and GLP-1 (1 µM), administered individually at subthreshold concentration and together. (D) Graph showing group data for responses to PYY and GLP-1 alone and in combination (n=7).

Mouse afferent recordings were performed to see if stimuli that activate EEC also activate afferent nerves from the same region and if convergence and synergism of signals were continued in this next step of signalling to the CNS. 5-HT_3_, GLP-1 and Y_2_ receptors are expressed on vagal afferent fibres and mediate nutrient-related signals from the gut to the central nervous system (see also online supplemental figure 3B).^32–35^ Vagal afferents play a key role in initiating satiety.^13 35^ To test the hypothesis that nutrient signals are transduced into afferent signals in the proximal colon by L cell and EC mediators that are located in close proximity to afferent nerves stained with calretinin—a putative vagal afferent marker (online supplemental figure 3)^36^—we developed a novel mouse preparation to record afferent activity. Using this model, we studied if convergence and summation occurred with nutrient-evoked afferent responses as we had shown for EEC responses. When LA (25 mM) and TUG-891 (10 µM) were coadministered, there was evidence of cooperativity of responses (figure 5A, B) but not to the extent seen in EEC responses, suggesting amplification was mainly at the level of the upstream EEC; this is supported by the observation of LA increasing release of GLP-1 and PYY from mouse mucosal tissue (online supplemental figure 4). In another series of experiments, application of exogenous GLP-1 and PYY independently produced convergent effects on >90% of fibres (figure 5C); 23 single units were tested with GLP-1, 19 of which responded; 34 single units received PYY, 22 of which responded; and 27 single units were tested with 5-HT, 17 of which responded. The 5-HT_3_ receptor antagonist granisetron significantly inhibited the response to 5-HT (online supplemental figure 5). The Y_2_ receptor agonist CYM 9484 significantly inhibited the response to PYY, and the GLP-1 response was significantly inhibited by the GLP-1 receptor antagonist Exendin 3–39 (online supplemental figure 5).^37^ The responses of afferent units to intraluminal perfusion with LA (25 mM) were reproducible, but absent after preadministration of all three antagonists (online supplemental figure 6), but not after each antagonist was applied individually in separate experiments (not shown).

10.1136/gutjnl-2020-323219.supp4 Supplementary data

10.1136/gutjnl-2020-323219.supp5 Supplementary data

10.1136/gutjnl-2020-323219.supp6 Supplementary data

10.1136/gutjnl-2020-323219.supp7 Supplementary data

In a subset of recordings, multiple mediators were applied to the same mouse tissue. In units given both PYY and GLP-1, all (7/7) responded to both. The observation that individual single units are able to respond to each of these mediators indicated that receptors for each coexist on the same afferent endings (figure 5C). Furthermore, combination of threshold concentrations of PYY and GLP-1 evoked powerful responses that were maximal for each fibre (figure 5D), seen also in combination experiments with 5-HT (online supplemental figure 5), indicating that there was convergence and synergism at the level of the EEC and at the level of the afferent ending.

## Discussion

Using a translational approach, we demonstrate that human colonic EECs are a key molecular target for maximising peripherally mediated appetite regulation and offer a novel therapeutic avenue for limiting food intake. We found EECs express multiple nutrient sensing GPCRs and that selective stimulation of GPR84 and FFAR4 in vitro results in activation of separate intracellular pathways leading to synergistic release of anorectic hormones PYY and GLP-1. These hormones may then activate peripheral nerve endings to increase neuronal activity. Importantly, in a proof-of-concept clinical trial in obese volunteers, we demonstrated that co-administration of GPR84 and FFAR4 agonists led to reduced food intake and increased levels of circulating PYY.

The EEC is becoming a key target in metabolic disease because of its role in orchestrating downstream responses in a range of neural, epithelial, endocrine and immune cells.^38–40^ Activation of EECs has long been the focus of several successful clinical strategies to prolong or boost the action or release of GLP-1 and PYY.^5 6^ We show that colonic EEC are equipped with multiple receptors for nutrients endogenous and exogenous to the colon, which together can trigger a potent endocrine response. Here we have described only two phosphoprotein pathways. These were mainly distinct to the particular cell studied, and there are likely to be several more.^41^ Intracellular pathways linking nutrient and metabolite sensing GPCR with peptide release are poorly understood, especially in EEC. Further research is required to understand allosteric interactions, G-protein coupling and cytoskeletal processes in exocytosis. Additionally, as we have previously reported,^18^ multiple GPCR activation results in entrainment of colonocytes and paracrine effects mediated by Y_1_,^42^ GLP-1^43^ and 5-HT_3_ receptors expressed on colonocytes.^44^ The paracrine effects observed may result in changes to absorption,^45^ immune cell/cytokine activity^46^ and crypt proliferation,^43^ some of which may have influenced food intake in this study.

Our data show that nutrient mediated processes continue downstream of the EEC, with afferent nerves from the colon also showing convergent responses to EEC mediators GLP-1 and PYY acting on GLP-1 and Y_2_ receptors, respectively. Several studies show afferent endings innervate the mucosal epithelium and terminate in close apposition to EECs.^27 47–49^ Small intestinal vagal afferents respond to CCK and 5-HT among others in a convergent way in some species,^32^ but not others.^50^ As PYY is the major EEC mediator in the lower gut,^51^ we sought to demonstrate convergence of PYY signalling relative to other mediators. The degree of convergence between EEC mediator-evoked responses suggested concurrent expression of 5-HT_3_, Y_2_ and GLP-1 receptors on afferent endings and is supported by studies that have shown the receptors exist on vagal afferents.^33 34 49^ We observed that these receptors acted super-additively to activate colonic afferents, which follows since they comprise both ion channel and G-protein coupled receptors with distinct downstream pathways. Therefore, afferent nerves can convey detailed information about the immediate epithelial hormonal environment to the CNS, providing a higher resolution map to the CNS than hormones arriving via the circulation.

We have shown two clear levels of convergence in this system: the first within the EEC itself, and the second in action of EEC mediators on afferent endings that subsequently signal to the central nervous system. Our proof-of-concept trial, mirroring findings from earlier ex vivo tissue experiments, demonstrated both reduced food intake and increased circulating levels of PYY. Other approaches to nutrient-induced changes to appetite include colonic delivery of short chain fatty acid propionate, which also reduces food intake^52^ and increases circulating levels of PYY and GLP-1, in acute and long-term administration studies.^23 46^ LA delivery leads to a small increase in serum GLP-1 levels.^53^ Both of these studies reported increased circulating levels of GLP-1 following nutrient delivery. This contrasts with the current trial, where GLP-1 levels were not significantly altered, yet calorific intake was lower in active versus placebo. This would suggest that circulating PYY predominates in mediating these effects. Alternatively, GLP-1 may be having a peripheral, locally mediated effect on GLP-1R receptors expressed on nearby mucosal afferent endings. This is further implicated by our electrophysiology data in mice showing potent local GLP-1R mediated effects. Finally, it is unsurprising that ghrelin levels were not significantly altered between active and placebo groups in the present study, as ghrelin is released primarily from the stomach, and the capsules used in the study were coated specifically for colonic release.

In our obese volunteer study, there was no effect of MCFA capsules on subjective hunger ratings but modest yet significant effects on energy intake. This reduction in calorific intake of approximately 12% could result in weight loss of 12 kg over 24 weeks (based on US National Institute of Health Guidelines)^54^ but requires validation in a long-term weight loss study. Additionally, unchanged subjective hunger ratings suggest a subliminal effect on appetite, which is a desirable feature of any obesity treatment, and one that we shall aim to retain in further larger scale clinical studies. We would speculate that this is a feature of stimulating the colon rather than the small intestine, which is a major source of signals that induce nausea, fullness and similar conscious perceptions. The data presented here warrant a chronic administration study with a larger number of volunteers to assess end-points of weight loss, examine hormone expression (over months) and assess population variability to treatment.

There are a number of limitations within the current study. For example, the absence of potent synthetic agonists for GPR84. We have used LA as it binds to the GPR84 to activate the receptor; however, this agonist may bind to other long-chain receptors. In addition, electrophysiology experiments in human colonic tissue would add a further translational advantage, while similar experiments in animal models of obesity and altered diet would provide insight to neuronal signalling pathways—these will be investigated in future studies. A clear limitation of the clinical trial using an acute administration paradigm is the lack of longer time-points for measurement of hormone release and effect on appetite perception. The capsule coating ensures colonic delivery, but this requires between 3 and 4 hours from ingestion to disintegration in the colon resulting from normal gastrointestinal motility time (as described in the Methods). As the active and placebo capsules are given over two points in a single day, it was not feasible to assess volunteers for more than 8 hours. Finally, although only CCK and ghrelin have been shown to be altered by oestradiol^55^ and despite inclusion of a 4-week washout to account for cycle phase of female volunteers, we cannot completely discount effect of hormonal variation in the results.

In conclusion, we demonstrate that specific targeting of multiple nutrient-sensing receptors stimulates convergent activation of EEC and colonic afferent endings. Importantly, we show that targeting colonic L cells by costimulating specific receptors in obese volunteers has the capacity to reduce food intake and boost PYY levels, thereby supporting our preclinical findings and offering the potential development of a novel and effective obesity treatment without the drawbacks of surgery. With these principles in mind, we can devise more appropriate strategies for targeting nutrient sensing in the colon for the benefit of treatment of obesity and type 2 diabetes and move on to larger scale studies including body weight regulation.

## Data Availability

All data relevant to the study are included in the article or uploaded as supplementary information.
