# Supplementary material for: Decoy bypass for appetite suppression in obese adults: role of synergistic nutrient sensing receptors GPR84 and FFAR4 on colonic endocrine cells
Source: Gut. 2021 Jun 3;71(5):928–37. doi: 10.1136/gutjnl-2020-323219 (PMC8995825; doi:10.1136/gutjnl-2020-323219)
Supplement: Supplementary data [file gutjnl-2020-323219supp001.pdf]

SUPPLEMENTARY METHODS

Primary Antibodies

GPR84 (Rabbit, Catalogue no: AGR-052, Alomone Labs, 1:200)

FFAR4 (Mouse, Catalogue no: sc-390752, Santa Cruz Biotechnology, 1:200)

PYY (Mouse, Catalogue No: NBP1-05167, Novus Biologicals, 1:200)

GLP-1 (goat, Catalogue no: SC-7782, Santa Cruz Biotechnology, 1:200)

5-HT (Rat, Catalogue no: ab22047, Abcam, 1:400)

pERK (Rabbit, Catalogue no: 4370, Cell Signalling, 1:200)

pCaMKII (Rabbit, Catalogue no: V1111, Promega, 1:200)

Nutrient solutions

Nutrients were made up in low-glucose Krebs solutions: 124.05mM NaCl (Sigma-Aldrich), 4.78mM KCl (Sigma-Aldrich), 1.33mM NaH2PO4 (Sigma-Aldrich), 2.44mM MgSO4 (Sigma-Aldrich), 5.50mM D-glucose (Sigma-Aldrich) and 25.00mM NaHCO3 (Sigma-Aldrich) and carbogenated with 95% O_2 and 5% CO_2. Lauric acid (25 mmolL^{-1}, Sigma-Aldrich) was used as an agonist of GPR84[26] and TUG891 (10 µM, Tocris Bioscience) used as specific and potent synthetic agonist of FFAR4[27].

Tissue dissection for electrophysiological recordings

Experiments were performed using male C57BL/6 mice (10-12 weeks of age) and in accordance with the UK Animal (Scientific Procedures) Act 1986. We developed a novel method of whole nerve recording of mouse proximal colon. A section of the alimentary canal comprising a short segment of ileum, the caecum, and the first 3cm of the proximal colon was cut from the abdominal viscera together with the mesentery, along the superior mesenteric artery where extrinsic afferents innervating the proximal colon travel to the superior mesenteric/coeliac ganglion (SMG/CG) complex. The ileocecal junction was ligatured and

most of the caecum removed. The lumen was washed free of faecal matter with Krebs solution before being placed in a bespoke tissue bath. The proximal colon was then cannulated for intraluminal perfusion - proximally (inflow) around the remnant caecum-colon junction and distally (outflow) along the proximal colon 3cm caudally.

Electrophysiological protocols

For all electrophysiological experiments, a minimum of 15 minutes of baseline spontaneous activity was recorded prior to conducting any experimental protocol. The tissue was perfused both extraluminally (6 ml/min; 32–34°C) and intraluminally (0.1 ml/min) with carbogenated (95% O_2, 5% CO_2) Krebs buffer (in mM: 124 NaCl, 4.8 KCl, 1.3 NaH_2PO_4, 2.5 $CaCl_2$, 1.2 $MgSO_4$.7H2O, 11.1 glucose, and 25 $NaHCO_3$) supplemented with atropine (10 μM), nifedipine (10 μM), and indomethacin (3 μM). The luminal Krebs perfusion was switched to lauric acid (25mM) and TUG891 (10μM), or combination to a total of 1mL after which the luminal perfusion solution was switched back to Krebs. There was a washout period of 45-60 min prior to a second addition of the nutrient solution. The preparation was continually perfused for viability, but this also served as our control for determining baseline discharge prior to switching the inlet to the nutrient or hormone solution. In a separate series of experiments, the receptor agonists were applied individually in a similar manner to examine proximal colonic afferent responses to exogenous 5-HT (300 μM), GLP-1 (1μM) or PYY (1μM). To examine the effect of blocking 5-HT_3, Y_2 and GLP-1 receptors during nutrient or agonist perfusion, the combination of granisetron (1μM), CYM-9484 (1μM), and exendin 9-39 (100nM) was applied prior to the second luminal perfusion of nutrient. Receptor antagonists were applied in a 'step-up' manner such that the antagonists were added directly to the recording bath to immediately achieve the above concentrations, followed by continuous superfusion of the antagonists into the bath for 15 min (140 mL) prior to nutrient perfusion.
