# Supplementary material for: Decoy bypass for appetite suppression in obese adults: role of synergistic nutrient sensing receptors GPR84 and FFAR4 on colonic endocrine cells
Source: Gut. 2021 Jun 3;71(5):928–37. doi: 10.1136/gutjnl-2020-323219 (PMC8995825; doi:10.1136/gutjnl-2020-323219)
Supplement: Supplementary data [file gutjnl-2020-323219supp002.pdf]

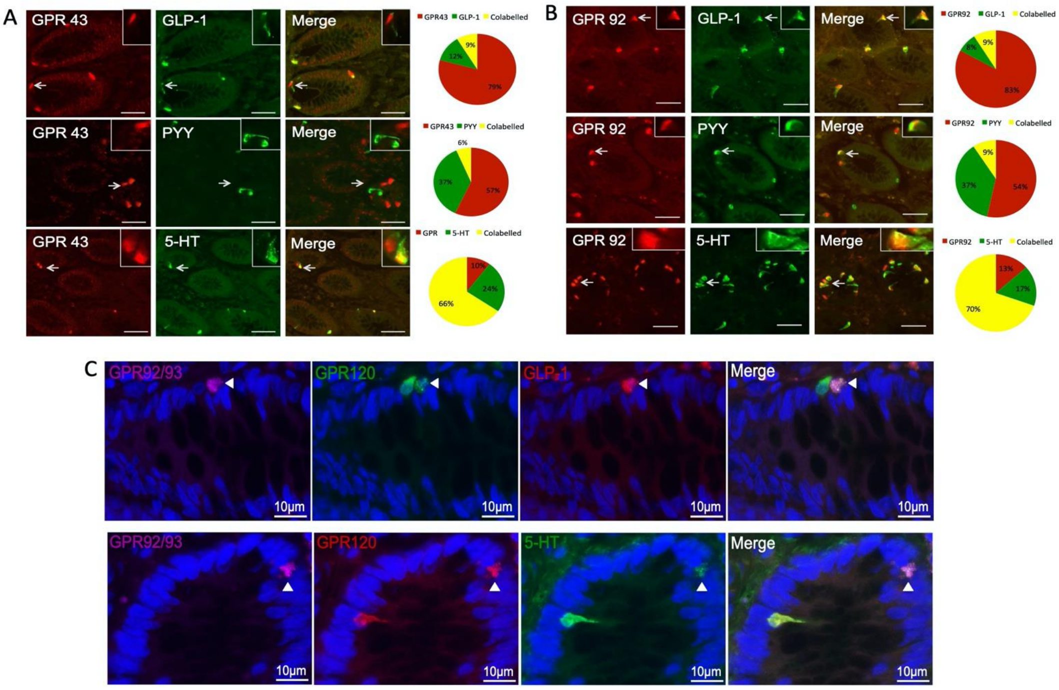

Supplementary Figure 1: L-cells and EC cells co-express short chain fatty acid and amino acid sensing GPCRs.

A. GPR43, a short chain fatty acid receptor, is expressed on both L- and EC cells in the human colon, with the majority of expression being on EC cells.

B. GPR92, peptone receptor, is similarly expressed on both EEC cell types, with 70% of EC cells displaying immunoreactivity.

C. GPR92 is co-expressed with FFAR4 on GLP-1 containing L-cells and 5-HT containing EC cells of the human colon.
