# Supplementary material for: Decoy bypass for appetite suppression in obese adults: role of synergistic nutrient sensing receptors GPR84 and FFAR4 on colonic endocrine cells
Source: Gut. 2021 Jun 3;71(5):928–37. doi: 10.1136/gutjnl-2020-323219 (PMC8995825; doi:10.1136/gutjnl-2020-323219)
Supplement: Supplementary data [file gutjnl-2020-323219supp003.pdf]

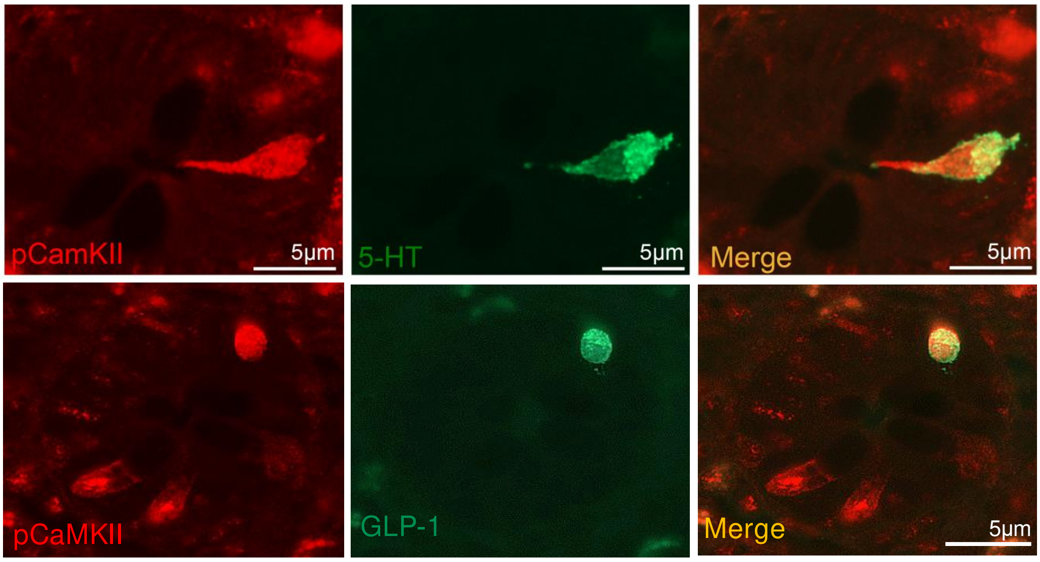

Supplementary Figure 2: Stimulation with FFAR4 TUG891 induces pCaMKII expression in GLP-1 containing L-cells and 5-HT containing EC cells.

A. Human colonic mucosa stimulated with TUG891 (10 μM) leads to co-expression of pCaMKII and EC cells.

B. Human colonic mucosa stimulated with TUG891 (10 μM) leads to co-expression of pCaMKII and L-cells.
