# Supplementary material for: Decoy bypass for appetite suppression in obese adults: role of synergistic nutrient sensing receptors GPR84 and FFAR4 on colonic endocrine cells
Source: Gut. 2021 Jun 3;71(5):928–37. doi: 10.1136/gutjnl-2020-323219 (PMC8995825; doi:10.1136/gutjnl-2020-323219)
Supplement: Supplementary data [file gutjnl-2020-323219supp004.pdf]

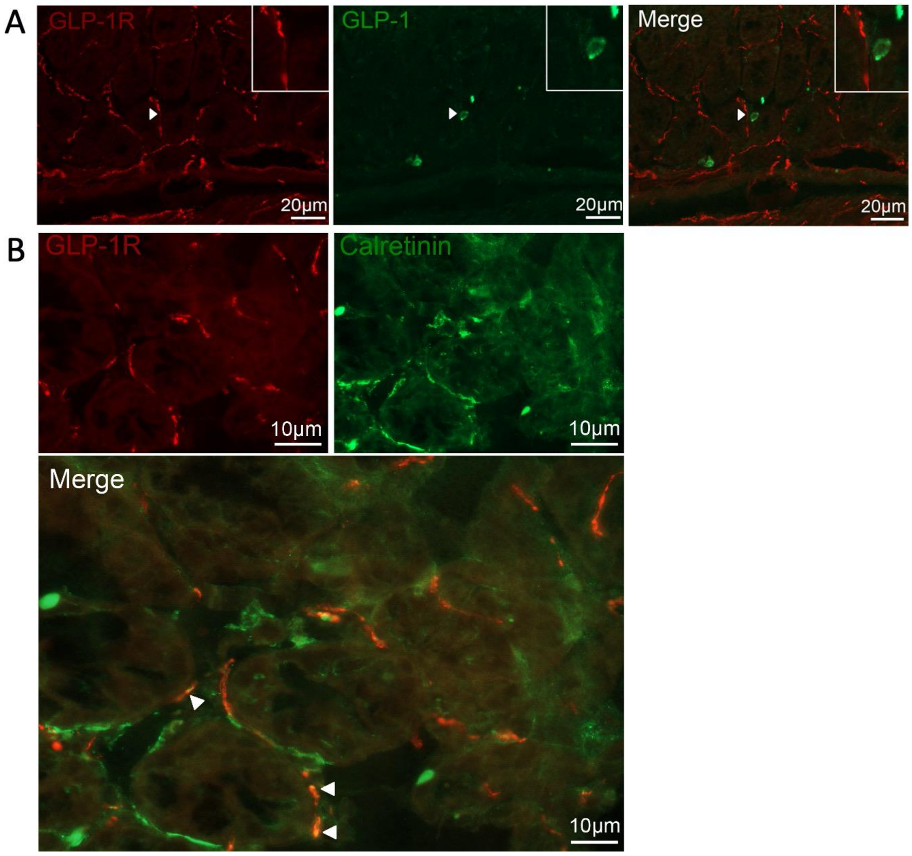

Supplementary Figure 3: GLP-1 positive L-cells are closely associated with GLP-1 receptor (GLP-1R) expressed on calretinin positive neurons.

A. GLP-1R immunoreactivity is found in close apposition to GLP-1 containing L-cells in the mouse proximal colon.

B. Calretinin (putative vagal afferent marker) positive nerve endings co-stain with GLP-1R (as shown by white arrows).
