# Supplementary material for: Decoy bypass for appetite suppression in obese adults: role of synergistic nutrient sensing receptors GPR84 and FFAR4 on colonic endocrine cells
Source: Gut. 2021 Jun 3;71(5):928–37. doi: 10.1136/gutjnl-2020-323219 (PMC8995825; doi:10.1136/gutjnl-2020-323219)
Supplement: Supplementary data [file gutjnl-2020-323219supp005.pdf]

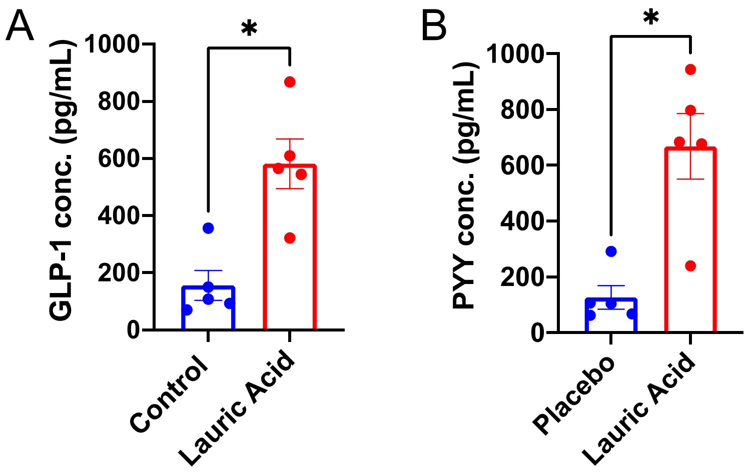

Supplementary Figure 4: GPR84 agonist lauric acid increases release of GLP-1 and PYY from mouse explants.

A. Stimulation of mouse explant tissue with lauric acid (25 $mmol^{-1}$) significantly increases GLP-1 release compared to stimulation with buffer control. N=5/group.

B. Stimulation of mouse explant tissue with lauric acid (25 $mmol^{-1}$) significantly increases PYY release compared to stimulation with buffer control. N=5/group.
