# Supplementary material for: Decoy bypass for appetite suppression in obese adults: role of synergistic nutrient sensing receptors GPR84 and FFAR4 on colonic endocrine cells
Source: Gut. 2021 Jun 3;71(5):928–37. doi: 10.1136/gutjnl-2020-323219 (PMC8995825; doi:10.1136/gutjnl-2020-323219)
Supplement: Supplementary data [file gutjnl-2020-323219supp006.pdf]

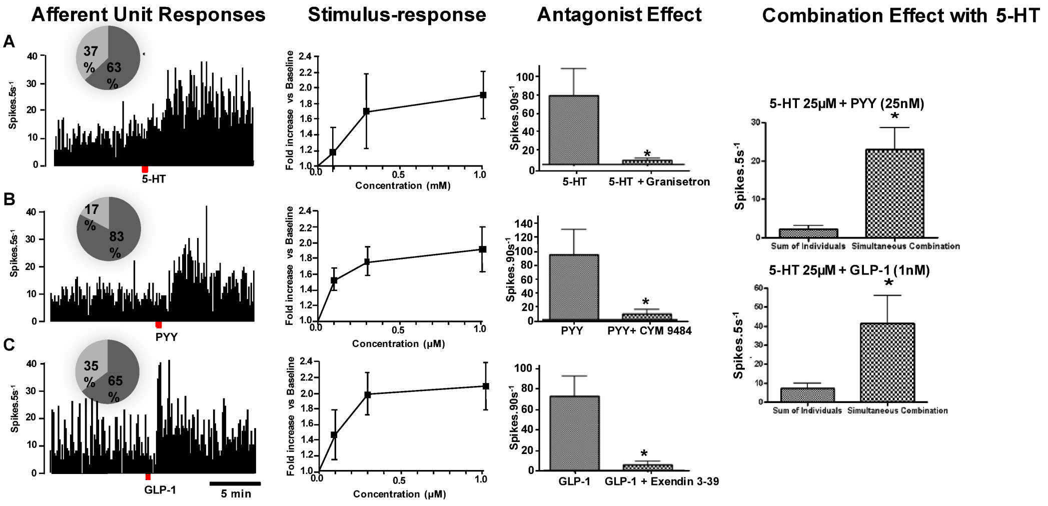

Supplementary Figure 5: 5-HT, PYY and GLP-1 – evoked responses in individual afferent fibres.

A. The 5-HT_3 receptor antagonist Granisetron (1µM) significantly inhibits response to 5-HT (300µM): N=7, *p*=0.035.

B. The Y_2 receptor antagonist CYM 9484 (2µM) significantly inhibits response to PYY 300nM. N =6, *p*=0.04.

C. GLP-1 response was significantly inhibited by the GLP-1 receptor antagonist Exendin 3-39 (100nM). N=8, *p*=0.04.

Responses to these agonists were concentration-dependent, and subthreshold concentrations of PYY (25nM) or GLP-1 (25nM) administered simultaneously caused significant activation compared to the individual responses added together (N=7, *p*=0.006 and 0.04, respectively).
