# Supplementary material for: Decoy bypass for appetite suppression in obese adults: role of synergistic nutrient sensing receptors GPR84 and FFAR4 on colonic endocrine cells
Source: Gut. 2021 Jun 3;71(5):928–37. doi: 10.1136/gutjnl-2020-323219 (PMC8995825; doi:10.1136/gutjnl-2020-323219)
Supplement: Supplementary data [file gutjnl-2020-323219supp007.pdf]

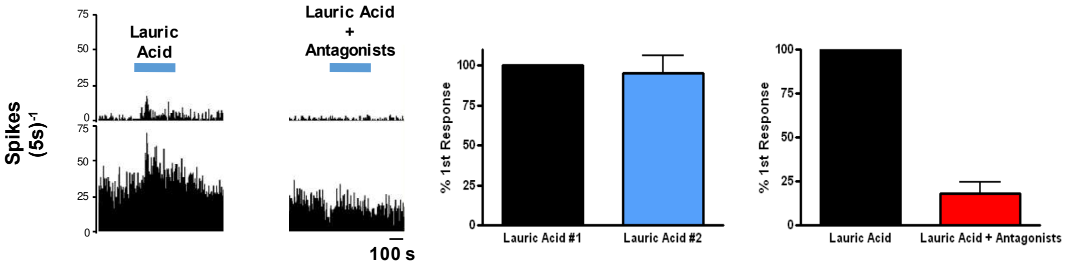

Supplementary Figure 6: Afferent excitation by lauric acid is blocked by a combination of antagonists.

Responses of afferent units to intraluminal perfusion with LA (25mM) are reproducible on second presentation, but absent after pre-administration of all three antagonists together with final concentrations - Granisetron (1µM), CYM 9484 (2µM) and Exendin 3-39 (100nM), (p=0.05, N=4-10).
